# Gut Microbiome as a Possible Cause of Occurrence and Therapeutic Target in Chronic Obstructive Pulmonary Disease

**DOI:** 10.4014/jmb.2301.01033

**Published:** 2023-03-30

**Authors:** Eun Yeong Lim, Eun-Ji Song, Hee Soon Shin

**Affiliations:** 1 Food Functionality Research Division, Wanju-gun, Jeollabuk-do 55365, Republic of Korea; 2 Department of Food Biotechnology, Korea University of Science and Technology, Daejeon 34113, Republic of Korea

**Keywords:** Gut microbiota, chronic obstructive pulmonary disease, short-chain fatty acids, probiotics, prebiotics, fecal microbiota transplantation

## Abstract

As a long-term condition that affects the airways and lungs, chronic obstructive pulmonary disease (COPD) is characterized by inflammation, emphysema, breathlessness, chronic cough, and sputum production. Currently, the bronchodilators and anti-inflammatory drugs prescribed for COPD are mostly off-target, warranting new disease management strategies. Accumulating research has revealed the gut–lung axis to be a bidirectional communication system. Cigarette smoke, a major exacerbating factor in COPD and lung inflammation, affects gut microbiota composition and diversity, causing gut microbiota dysbiosis, a condition that has recently been described in COPD patients and animal models. For this review, we focused on the gut–lung axis, which is influenced by gut microbial metabolites, bacterial translocation, and immune cell modulation. Further, we have summarized the findings of preclinical and clinical studies on the association between gut microbiota and COPD to provide a basis for using gut microbiota in therapeutic strategies against COPD. Our review also proposes that further research on probiotics, prebiotics, short-chain fatty acids, and fecal microbiota transplantation could assist therapeutic approaches targeting the gut microbiota to alleviate COPD.

## Introduction

The human gastrointestinal tract harbors approximately 10^14^ microorganisms [1]. The gut microbiota, known as the second genome, plays key roles in host biological processes such as nutrient [2] and drug [3] metabolism, defense against pathogens [4], and maintenance of gut barrier function [5] and immune homeostasis [6, 7]. The most prevalent bacteria in the human gut are Firmicutes, Bacteroidetes, Proteobacteria, Actinobacteria, and Verrucomicrobia, which together account for over 90% of the gut microbial community [8]. Of these, the most common phyla, Firmicutes and Bacteroidetes, have a major influence on maintaining host health.

A healthy gut microbiota is characterized by diversity, stability, resilience, and symbiosis with the host [9]. However, exposure of the host to various factors such as antibiotics, poor diet, infection, and smoking, can cause an imbalance known as dysbiosis in the structure of the gut microbiota [10-14]. Antibiotic use reduces alpha diversity in the gut microbiota [10, 11]. Intervention with a Western diet for 7 months can cause gut dysbiosis, as indicated especially by decreased *Bifidobacterium* spp. in mice [12]. In addition, intranasal inoculation of the SARS-CoV-2 virus induces gut microbiota dysbiosis in mice, causing them to exhibit increased relative abundances of *Akkermansiaceae*, Proteobacteria, and *Escherichia-Shigella* [13]. Smokers reportedly have a higher abundance of Bacteroidetes, with decreased Firmicutes and Proteobacteria compared with non-smokers [14].

Gut microbiota dysbiosis can be related to gastrointestinal as well as brain, liver, and lung diseases [15]. Recently, there have been reports that lung diseases, such as COPD, asthma, lung cancer, and lung fibrosis are associated with gut microbiota dysbiosis [16]. Notably, COPD is a severe, persistent airway and lung disease; the effectiveness of current treatments is limited and warrants the need for further research on potential therapeutic options [17-19]. This review summarizes the findings of existing research on the association between gut microbiota dysbiosis and the etiology or pathogenesis of COPD, as well as the regulation of the gut microbiota for COPD treatment.

## Gut–Lung Axis

The gut microbiome interacts by establishing an axis between several organs, including the brain, kidney, liver, bone, and heart [20]. Several studies have proposed the gut–lung axis to be a bi-directional connection between the gut and lungs. Approximately 50% of adult patients with inflammatory bowel disease (IBD) and 33% of patients with irritable bowel syndrome (IBS) also showed respiratory symptoms, such as dyspnea, cough, asthma attacks, and waking up at night due to shortness of breath or coughing during the previous year [21, 22]. Additionally, patients with very severe COPD or uncontrolled asthma show a higher gastrointestinal symptom score than patients with mild COPD or well-controlled asthma, suggesting that the severity of lung disease is associated with the severity of gut disease [23]. Moreover, gut microbiota-depleted mice are more susceptible to lung infections, pointing to a protective role of the gut microbiota [24, 25]. Interestingly, bacterial and viral respiratory infections in the lungs of mice lead to gut microbiota dysbiosis [26-28]. In this section, we review the communication of the gut–lung axis, focusing mainly on the gut microbial metabolites, bacterial translocation, and immune cell modulation via mesenteric lymph nodes (MLNs) and bloodstream.

## Gut Microbial Metabolites

Microbiota-accessible carbohydrates (MACs) are complex plant carbohydrates that cannot be completely absorbed in the small intestine, and reach the large intestine for use by microorganisms [29]. Short-chain fatty acids (SCFAs), such as butyrate, acetate, and propionate are major metabolic products of MAC fermentation [29]. SCFAs exert beneficial effects on energy metabolism, gut barrier function, and immune regulation [30]. Moreover, SCFAs enhance gut barrier integrity by increasing mucin production and upregulating the expression of tight junction protein [31-35]. In particular, butyrate has been found to upregulate the expression of claudin-1, claudin-3, claudin-4, occludin and ZO-1 [33-35], while downregulating the expression of claudin-2 to strengthen the epithelial barrier integrity in epithelial cells [32]. Sodium butyrate improved the gut permeability and increased protein expression of mucin-2 protein, as well as the mRNA expression of tight junction proteins through the activation of GPR109A in a 2,4,6-trinitrobenzene sulfonic acid-induced mouse IBD model [36]. SCFAs regulate the activation, recruitment, and differentiation of immune cells, including neutrophils, macrophages, dendritic cells, and T lymphocytes. They are also associated with various lung diseases such as COPD, and asthma [37-39]. In addition, SCFAs are involved in airway epithelial dysfunction. Butyrate and propionate treatment restored airway epithelial dysfunction and increased the expression of ZO-1 in human bronchial epithelial cells 16HBE14o- [40]. The airway epithelial barrier dysfunction is closely associated with COPD pathogenesis, and therefore, SCFAs may help alleviate the symptoms of this disease.

## Bacterial Translocation

Bacterial translocation is the process by which bacteria and bacterial products transit from the gastrointestinal tract to extra-intestinal sites, including the MLNs, bloodstream, and distant organs [41]. In a healthy state, the epithelial cell layer and tight junction protein form the gut barrier to protect against antigens, pathogens, and toxins.

Impaired gut integrity increases the permeability of the gut barrier, facilitating bacterial translocation, which has been observed in several lung diseases [42, 43]. Exposure to cigarette smoke for 10 weeks (5 days a week) significantly increased the bacterial translocation rate in the MLNs [44]. Furthermore, lung microbiota in the sepsis model showed increased relative abundance in certain species belonging to the *Enterobacteriaceae* family and *Enterococcus faecalis*, which are normally present in the gut microbiota [45].

Lipopolysaccharide (LPS), a major component of gram-negative bacteria, is associated with the development of several diseases by increasing the permeability of the gut barrier [46, 47]. Germ-free mice showed relatively low LPS concentration in the lungs, but this is reversed by colonization with the gram-negative bacterium *Bacteroides thetaiotaomicron*, suggesting LPS translocation from gut to lung [48]. Mice receiving antibiotic treatment showed an impaired immune response to the respiratory influenza A virus, which was reversed by intrarectal LPS inoculation [49]. These studies suggest that bacterial products from bacterial translocation could affect immune responses via various regulatory mechanisms.

## Immune Cell Modulation

The gut microbiota modulates lung immunity by regulating innate and adaptive immune responses. An antibiotic-induced gut microbiota suppression model showed an altered immune response. Mice treated with antibiotics showed impaired function of lung dendritic cells, which then exhibit low macrophage-inducible C-type lectin expression [50]. Dendritic cell dysfunction contributes to the increased susceptibility to *Mycobacterium tuberculosis* infection as a result of decreased activation of naïve CD4^+^ T cells. Another study showed that the administration of antibiotics induced gut microbiota dysbiosis, which causes immunosuppression in the lung and depresses dendritic cell bone marrow progenitors, resulting in the aggravation of lung infection caused by *Pseudomonas aeruginosa* [51]. Intrarectal inoculation of LPS reversed the antibiotic-induced impairment of immune responses, resulting in increased inflammasome activation and dendritic cell migration to the MLNs following intranasal influenza A virus infection [49].

The gut microbiota could modulate immune responses and alleviate lung diseases. Patients with nontuberculous mycobacterial (NTM) pulmonary disease and NTM-infected mice showed decreased levels of L-arginine in sera. Moreover, in NTM-infected mice compared to untreated mice, oral administration of L-arginine or fecal microbiota transplantation (FMT) from L-arginine-treated mice showed enhanced M1 macrophage and protective Th1 responses as well as alteration of gut microbiota, with increased relative abundance of *Bifidobacterium*, *Bilophila*, and unclassified YS2 and decreased relative abundance of *Odoribacter*, *Prevotella*, and *Akkermansia* [52]. Another study revealed that the administration of novel probiotic *Parabacteroides goldsteinii* MTS01 improves the symptoms of COPD by reducing activation of B cell signaling pathway and LPS activity in COPD mice [53].

## COPD and Gut Microbiota Dysbiosis

Cigarette smoking, a top risk factor for COPD, has been shown to alter gut microbiota in clinical and preclinical studies. Interestingly, about 30% of patients with COPD have no history of smoking, suggesting other possible risk factors for COPD [54]. Recently, dysanapsis, a mismatch of airway tree caliber to lung size, was named as a risk factor associated with COPD [55]. Gut microbiota is a possible risk factor for COPD etiology and progression, as gut microbiota dysbiosis has been identified in patients with COPD, and FMT from patients with COPD to mice aggravated lung function [37].

## COPD

COPD is a heterogeneous disease ranked as the third leading cause of death worldwide in 2019 [56]. COPD is characterized by chronic inflammation in the airways and lungs, with increased alveolar macrophages and neutrophils as well as respiratory symptoms including airway inflammation, emphysema, breathlessness, chronic cough, and sputum production [57]. The risk factors of COPD include cigarette smoking, exposure to secondhand smoke, air pollution, ambient particulate matter, and aging [58]. Furthermore, patients with COPD have an increased risk for comorbidities such as cardiovascular diseases, lung cancer, diabetes, metabolic syndrome, osteoporosis, anxiety, and depression [59]. COPD is classified by severity into four stages and different medications are prescribed for treatment based on the stage of progression [60].

Current treatment options for COPD include pharmacological and non-pharmacological strategies. Pharmacological interventions include bronchodilators, anti-inflammatory drugs, and antibiotics, whereas non-pharmacological interventions include pulmonary rehabilitation, oxygen therapy, and smoking cessation [61]. Until now, COPD treatment has been mainly symptomatic, and no agent can fundamentally cure COPD without side effects [18]. Therefore, there is an urgent need to elucidate the mechanism of COPD and explore novel treatment options.

## Association between Gut Microbiota and COPD 

Cigarette smoking induces compositional alterations in the gut microbiota [14, 62, 63]. Compared to the non-smokers, current smokers showed a reduced ratio of Firmicutes/Bacteroidetes (F/B), decreased relative abundance of the phyla Firmicutes and Proteobacteria and increased relative abundance of the phylum Bacteroidetes [14]. Another study revealed that the gut microbiota of smokers had a significantly lower relative abundance of Fusobacteria and Tenericutes compared to that of non-smokers [63]. At the species level, *Bacteroides thetaiotaomicron* and *Lactobacillus amylovorus* were increased, whereas *Dialister invisus* and *Ruminococcus bromii* were decreased in the gut microbiota of smokers. Moreover, patients with COPD exhibited gut microbiota dysbiosis [37, 64, 65]. The abundance of *Streptococcus*, *Rothia*, *Romboutsia*, and *Intestinibacter* was reported to be higher, whereas that of *Bacteroides*, *Roseburia*, and *Lachnospira* was lower in patients with COPD [64]. At the family level, *Bifidobacteriaceae*, *Eubacteriaceae*, *Lactobacillaceae*, *Micrococcaceae*, *Streptococcaceae*, and *Veillonellaceae* were enriched, whereas *Desulfovibrionaceae*, *Gastranaerophilaceae*, and *Selenomonadaceae*, along with several uncharacterized families of Bacilli and Clostridia, were depleted in patients. In addition, changes in the gut microbiota were observed according COPD stage [37, 65]. For example, the COPD III–IV group had lower levels of Bacteroidetes than the healthy or COPD I–II group, and higher levels of Firmicutes than the COPD I–II group [37]. The COPD I–II group had higher levels of *Prevotella*ceae, whereas the COPD III–IV group had lower levels of *Bacteroidaceae* and *Fusobacteriaceae* than the healthy group. Several preclinical models have shown gut microbiota dysbiosis in COPD. For example, the COPD group has significantly lower microbial richness and gut microbiota dysbiosis in rat and mice models [66, 67], and exhibits a lower relative abundance of *Allobaculum*, *Tyzzerela_3*, *Akkermansia*, and *Subdoligranulum*, which were positively correlated with body weight and lung function but negatively associated with the T helper 17 (Th17)/T regulatory cell (Treg) ratio and inflammatory cytokines in the lung [67]. A higher relative abundance of *Streptococcus*, *Marvinbryantia*, and *Candidatus_Stoquefichus* compared to that in normal mice is negatively associated with body weight and lung function but positively correlated with Th17/Treg balance and pro-inflammatory cytokines [67]. Disparities exist between preclinical and clinical studies, which may be due to the limitation of animal models, which cannot accurately reflect the different stages of COPD [68].

FMT was used to confirm the causal direction from the gut microbiota to COPD. Mice with FMT from COPD III–IV patients showed COPD-associated symptoms and immune responses such as weight loss, high plasma inflammatory cytokines, BALF immune cell infiltration, airway remodeling, and mucus hypersecretion [37]. Moreover, biomass fuel smoke exposure-induced COPD mice with FMT from patients with COPD for 28 days showed aggravated lung function compared to the COPD mice which did not receive the FMT [37]. These findings confirmed that gut microbiota is a causal factor for COPD. In contrast, FMT from healthy mice to COPD model mice alleviated disease symptoms such as emphysema development and lung inflammation while improving lung function [38, 53]. Collectively, gut microbiota could be a factor in the development and treatment of COPD.

## Therapeutic Strategies for COPD 

Homeostasis in gut microbiota is one of the potential considerations for the prevention or treatment of COPD. Dietary supplementation with probiotics, prebiotics, and SCFAs are known to improve gut microbiota homeostasis, maintain gut barrier integrity, enhance immune function, and exert beneficial effects in lung diseases. In addition, FMT is one of the potential therapeutic strategies to regulate COPD symptoms by the modulation of gut microbiota. In the following sections, we will describe several therapeutic strategies for COPD by restoring the gut microbiota using probiotics, prebiotics, SCFAs, and FMT.

## Probiotics

Probiotics are living microorganisms that provide important health benefits to the host when administered in sufficient quantities [69, 70]. In general, probiotics can defend the host against pathogens by regulating luminal pH, competing for adhesion sites, and producing antimicrobial peptides [71]. In particular, probiotics enhance gut barrier function by producing SCFAs and by upregulating the expression of tight junction proteins, such as claudin-1, occludin, ZO-1, and ZO-2 [30, 72]. Furthermore, probiotics affect the immune system via the production of cytokines and stimulation of immune cells [73]. Administration of probiotics has been shown to improve the pathogenesis of immune and metabolic diseases, including obesity, diabetes, and IBD [74-76].

Probiotics are promising targets for use in preventing and alleviating respiratory diseases, including respiratory tract infections, asthma, and cystic fibrosis [77-79]. *Bifidobacterium animalis* subsp. *lactis* Bl-04 supplementation for 28 days reduced the nasal lavage virus titer as well as the percentages of virus shedding in nasal secretions in the volunteers compared to the placebo group [77]. Co-administration of *Bifidobacterium lactis* Probio-M8 supplementation (3×10^10^ CFU/day) with conventional therapy Symbicort Turbuhaler synergistically improved asthma-related symptoms, including lower levels of fractional exhaled nitric oxide and alveolar nitric oxide, and higher asthma control test score than conventional therapy [78]. Furthermore, *Lactobacillus rhamnosus* GG administration for 1 month to children with cystic fibrosis reduced fecal calprotectin, a biomarker of cystic fibrosis, and recovered gut microbiota dysbiosis [79].

Administration of *L. rhamnosus* for COPD treatment (7 days prior to the COPD induction; thrice a week for 8 weeks) attenuates the inflammatory response and downregulates the expression of transcription factors, such as NF-κB and STAT3 in cigarette smoking-induced COPD mouse model [80]. Restoring gut microbiota dysbiosis via the enrichment of depleted microorganisms in disease models may be a significant probiotic target for the attenuation of COPD. For example, administration of *P. goldsteinii*, decreased in cigarette smoking-induced COPD symptoms including weight loss, infiltration of inflammatory cells, increased inflammatory gene expression and improved lung function [53]. This evidence suggests that the administration of probiotics based on gut microbiota composition can be an effective therapeutic strategy for improving COPD symptoms.

## Prebiotics

Prebiotics are indigestible food components that may produce beneficial effects by selectively stimulating the growth and/or activity of certain types of bacteria in the colon, to improve the health of an individual [81]. Prebiotics include dietary fibers and natural sugars that stimulate the beneficial bacteria in the gut and modulate the gut microbiota composition [82]. In particular, prebiotics could modulate gut barrier function, immune responses mediated by immune cells, and epithelial cell function [83]. Furthermore, prebiotics exert beneficial effects on diarrhea, IBD, obesity, type II diabetes, and colorectal cancer [84].

Prebiotics and dietary fiber decrease the incidence of respiratory diseases [85-89]. Prebiotic supplementation (galactooligosaccharide and polydextrose mixture) showed a significantly lower incidence of respiratory tract infections compared with the placebo group [86]. Fructooligosaccharide and galactooligosaccharide treatment attenuated the inflammatory symptoms in the OVA-LPS-induced allergic asthma and acute airway inflammation in a mice model, resulting in decreased levels of cytokines, leukotrienes, as well as reduced gene expression of AKT, NLR3, NF-kB, and MyD88 [85].

A high-fiber (cellulose or pectin) diet for three weeks was shown to have a protective role against the progression of cigarette smoking-induced emphysema in mice [88]. High dietary fiber is also associated with better lung function and reduced prevalence of COPD [87]. In addition, the high-fiber diet leads to changes in bone marrow hematopoiesis, particularly by enhancing the generation of Ly6c^−^ patrolling monocytes in mice with influenza infection [90]. The number of macrophages having limited capacity to produce chemokine C-X-C ligand 1 (CXCL1) in the airways is increased, whereas neutrophil infiltration and tissue damage is decreased. In a prospective cohort study involving women, 10 years of high dietary fiber intake is negatively correlated with COPD risk [89]. Therefore, prebiotics could be a potential treatment option for patients with COPD.

## SCFAs

Oral administration of SCFAs or increased levels in the colon is associated with the attenuation of lung diseases, including allergic asthma, lung fibrosis, and COPD [91-93]. For example, one-year-old children with high fecal SCFAs (butyrate and propionate) showed less prevalence of atopic sensitization and development of asthma between the ages of 3 and 6 years [91].

Low levels of SCFAs are found in the particulate matter-induced COPD rat model in proximal colon contents [66]. Consistent with preclinical studies, the levels of acetic acid, isobutyric acid, and isovaleric acid were decreased in patients with COPD III–IV but not in the group with COPD I–II compared to the healthy group, suggesting a negative association between the severity of COPD and lower levels of SCFAs [37]. Increased levels of SCFAs are also associated with the attenuation of COPD symptoms. For example, animal models with high concentrations of SCFAs by FMT or a high-fiber diet could protect against lung inflammation and emphysema [38]. Oral administration of SCFAs (acetate, propionate, and butyrate) for 3 weeks can decrease the inflammatory response and emphysema in COPD mice. Although the mechanism and efficacy of SCFAs on COPD remain to be established by further studies, these results suggest that SCFA intake may confer a positive effect in COPD therapy.

Emerging studies have shown that SCFA supplementation modulates the immune response in respiratory diseases. Butyrate supplementation with drinking water reduced the expression of neutrophil-attracting chemokine CXCL1 in lung macrophages, resulting in prolonged survival and reduced clinical score against influenza infection [90]. Butyrate and propionate treatment inhibited IgE/antigen-induced mast cell degranulation by modulation of HDAC activity in human and mouse mast cells, but not GPR41, GPR43, or peroxisome proliferator-activated receptors [94]. Notably, butyrate treatment downregulated the key genes of mast cell activation, such as Bruton’s tyrosine kinase (BTK), spleen tyrosine kinase (SYK), and Linker for Activation of T cells (LAT). Propionate treatment protects against allergic airway inflammation by enhancing dendritic cell hematopoiesis in the bone marrow, depending on G protein-coupled receptor 41, and the increased dendritic cell precursor is subsequently impaired in its ability to induce T helper type 2 cell differentiation in mice [95]. Acetate in drinking water also suppressed allergic airway disease in mice by promoting Treg numbers and function [96]. These results suggest that SCFAs alleviate COPD symptoms by regulating the immune response.

## FMT 

FMT is a method used to transfer microorganisms in stool samples from a donor to a recipient to directly alter the gut microbiota of the recipient [97] and normalize the gut microbiota composition. FMT is widely accepted as a safe and successful treatment for *Clostridioides difficile* infection [98].

Various studies also have suggested that FMT is effective in COPD. FMT from healthy mice to smoking- and poly I:C-induced COPD mice decreased the severity of emphysema, indicating decreased mean linear intercept and apoptosis [38]. COPD mice receiving FMT from normal mice had significantly ameliorated COPD symptoms, including body weight change, BALF cell infiltration, and lung function [38]. In addition, a combination of FMT and a high-fiber diet more potently attenuates lung inflammation. To date, there has been no clinical study on FMT in COPD, and further studies are needed. Future studies using FMT may provide important evidence for understanding the role of gut microbiota in COPD and its potential as a treatment strategy.

## Conclusion

COPD is a chronic lung disease with high morbidity. However, the paucity of safe and effective treatments warrants new treatment approaches to resolve COPD without side effects and severe sequelae. Emerging research has emphasized the role of the gut–lung axis in respiratory diseases, including allergic asthma, lung fibrosis, and COPD. To develop an effective therapy for COPD, consideration of the gut microbiota may be an effective approach. In this review, we discussed the gut–lung axis focusing primarily on the gut microbial metabolites, bacterial translocation, and immune cell modulation. We then summarized the association between the gut microbiota and COPD, finally suggesting probiotics, prebiotics, SCFAs, and FMT as promising therapeutic agents for COPD. Future studies are required to elucidate the exact mechanism and therapeutic efficacy of restoring gut microbiota in association with COPD as well as other inflammatory conditions of the lung.
